# Establishment of accurate estimation equations for area under the concentration curves using simultaneous limited sampling for extended-release tacrolimus and mycophenolic acid in kidney transplant recipients

**DOI:** 10.3389/fimmu.2026.1710261

**Published:** 2026-02-03

**Authors:** Kyoko Minamisono, Toshimasa Nakao, Takehiro Ohyama, Sho Nishida, Hajime Sasaki, Takayuki Hirose, Kiyohiko Hotta, Makiko Mieno, Daiki Iwami

**Affiliations:** 1 Division of Renal Surgery and Transplantation, Department of Urology, Jichi Medical University, Shimotsuke, Japan; 2 Department of Kidney Transplant Surgery, Sapporo City General Hospital, Sapporo, Japan; 3 Department of Renal and Genitourinary surgery, Faculty of Medicine, Hokkaido University, Sapporo, Japan; 4 Division of Center for Information, Jichi Medical University, Shimotsuke, Japan

**Keywords:** area under the curve, immunosuppressive drug, limited sampling strategy, prediction equations, renal transplantation

## Abstract

**Introduction:**

This study aimed to develop a limited sampling strategy (LSS) and predictive equations to accurately estimate the areas under the concentration-time curves (AUC) of extended-release tacrolimus (TAC-ER) and mycophenolic acid (MPA).

**Methods:**

A retrospective analysis of Japanese kidney transplant recipients yielded 90 TAC-ER AUC0-24 (23 patients) and 80 MPA AUC0-12 (29 patients) datasets, which were randomly split into learning and validation datasets. Training datasets were used to generate the LSS model equations based on multiple linear regression analysis, and the coefficient of determination (R2) was used to assess the goodness of fit of regression models. Validation datasets applied the selected training equations to compute error indices, Passing-Bablok’s Kendall’s τ, and Bland–Altman limits of agreement, thereby assessing predictive bias, accuracy, and precision.

**Results and discussion:**

Four equations (C0-C1-C6, C0-C1-C2-C6, C0-C1-C3-C6, C0-C1-C4-C6) showed strong correlations with the actual AUC (R² > 0.95), with the validation identifying C0-C1-C3-C6 as the most reliable for both TAC-ER and MPA. This study demonstrated that LSS using C0-C1-C3-C6 reliably and accurately estimated both the actual TAC-ER AUC0-24 and MPA AUC0-12 simultaneously in kidney transplant recipients. These equations can be feasibly implemented in outpatient clinical settings to reduce time and cost.

## Introduction

The optimization of drug dosing through therapeutic drug monitoring (TDM) is essential for improving graft and patient outcomes after kidney transplantation. Because immunosuppressive drugs used after kidney transplantation have a narrow therapeutic range, patients are prone to developing infections or renal toxicity due to excessive immunosuppression or rejection due to insufficient immunosuppression (1–3).

Trough concentration (C_0_), which is mostly used in clinical practice for TDM, may not accurately represent overall drug exposure in all cases, potentially leading to suboptimal dosing, especially for mycophenolate mofetil (MMF) (4–7). Area under the concentration-time curve (AUC) was informative utilized as one of the key pharmacokinetic parameters (8). Moreover, accurately determining AUC typically requires frequent blood sampling (often 8–10 times), which is impractical in clinical settings because of the burden on patients and associated costs. Limited sampling strategies (LSS) involving 3–5 blood samples have been proposed as valuable tools for accurately estimating the actual AUC while reducing the burden on patients.

Tacrolimus (TAC) and MMF are commonly co-administered in >90% of kidney transplant recipients (9–13). While many centers administer MMF at a fixed dose, numerous reports have highlighted the importance of monitoring drug exposure using AUC to achieve individualized therapy. Accordingly, MPA exposure is assessed via MPA-AUC in the United States, Europe, China, and Japan, following regional guidelines and recommendations (14–18). Similarly, therapeutic drug monitoring of TAC, particularly once-daily extended-release TAC (TAC-ER), remains essential to ensure adequate and personalized immunosuppressive therapy (9, 19).

However, to date, a common set of limited sampling points for simultaneously predicting the AUC of once-daily extended-release TAC (TAC-ER) and mycophenolic acid (MPA), the pharmacologically active metabolites of twice-daily MMF in kidney transplant recipients, has not been established. If the AUCs of both MPA and TAC-ER can be accurately estimated using a limited set of common sampling points, this approach could serve as an effective tool for precise and efficient monitoring of drug exposure in patients.

This study aimed to develop an LSS capable of simultaneously predicting the AUC of TAC-ER and MPA in kidney transplant recipients. Predictive equations for the actual AUC of TAC-ER and MPA, using common blood sampling points and maintaining high precision and accuracy, have the potential to contribute to the prevention of post-transplant complications such as infections and graft rejection.

## Materials and method

### Patients and protocols

This study included 108 Japanese kidney transplant recipients who underwent transplantation at the Hokkaido University or Sapporo City General Hospital. Between 2006 and 2010, these patients underwent a series of nine blood sample collections before and after drug administration to determine the actual AUC for TAC-ER or MPA.

**Figure 1:**
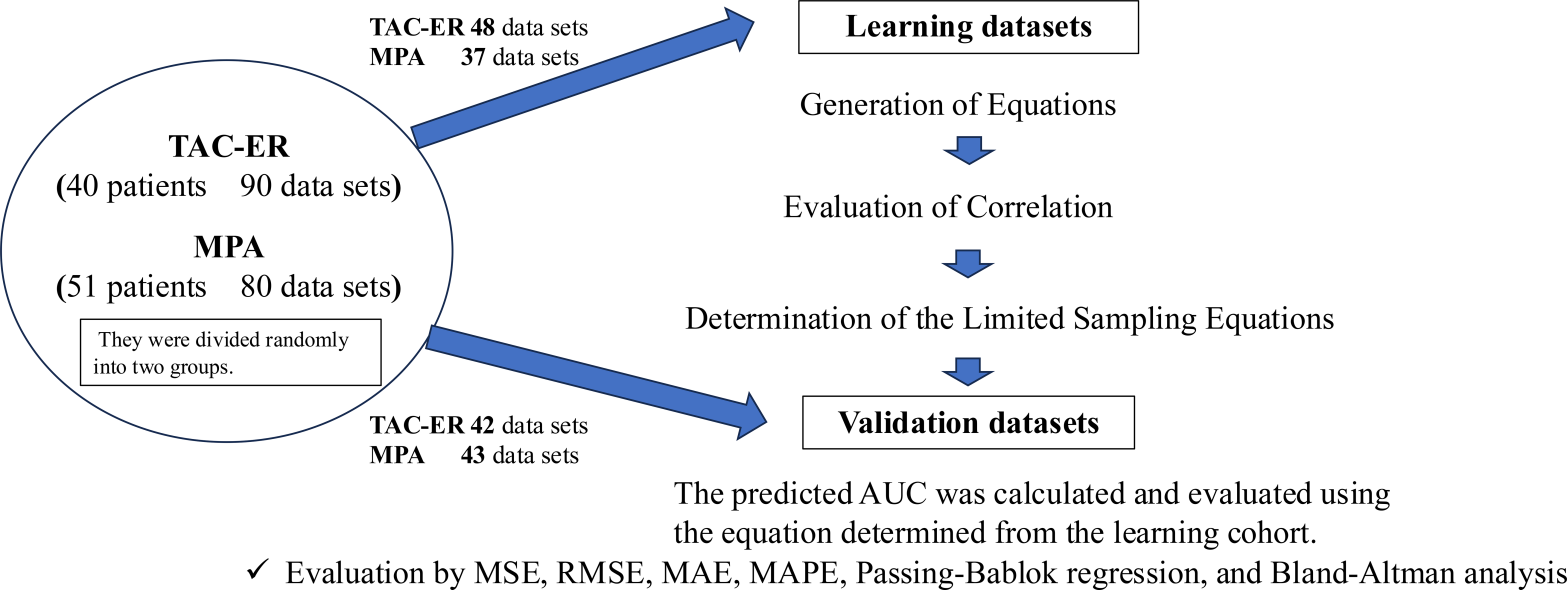
Flowchart for dividing the pharmacokinetic data of TAC-ER and MPA in kidney transplant recipients into learning and validation datasets. Pharmacokinetic data were obtained from 52 renal transplant recipients for TAC-ER and 29 recipients for MPA. Patients’ blood concentration data from nine sampling points were randomly divided into learning and validation datasets. For TAC-ER, 90 data points were assigned, while for MPA, 80 data points were included. The learning dataset was used to derive predictive equations for AUC based on 2–4 sampling points using linear regression. The validation dataset evaluated prediction precision using MSE, RMSE, MAE, and MAPE, Passing-Bablok regression, and the Bland–Altman analysis. TAC-ER, extended-release tacrolimus; MPA, mycophenolic acid; MSE, mean squared error; RMSE, root mean squared error; MAE, mean absolute error; MAPE, mean absolute percentage error; AUC, area under the curve;.

In this study, data were retrospectively extracted from past clinical records, with a particular focus on nine previously conducted blood sampling points to determine the actual AUC values. Of the 108 included individuals, retrospective data collection yielded 90 actual TAC-ER AUC data points from 57 patients after renal transplantation. Similarly, for the remaining 51 individuals, retrospective analysis provided 80 MPA AUC data points (**Figure 1**).

Using data from nine-point blood sampling (TAC-ER: pre-dose and post-dose at 0.5, 1, 2, 3, 4, 6, 12, and 24 h; MPA: pre-dose, and post-dose at 0.5, 1, 2, 3, 4, 6, 8, and12 hours), which has already been used in routine clinical practice for measuring TAC-ER or MPA blood concentrations to calculate actual AUC by trapezoidal method, we analyzed the pharmacokinetics of these drugs. The Jichi Medical University Hospital Bioethics Committee for Clinical Research approved the study protocol (approval number: CU23-172).

### Analysis

All the datasets were randomly split into two distinct datasets: learning and validation. Initially, multiple regression analysis was applied to the learning datasets to derive predictive equations for the actual AUC of TAC and MPA based on all possible combinations of blood concentrations at various sampling points. The actual AUC was calculated using the linear trapezoidal method. Subsequently, prediction equations were generated through linear regression analysis using all possible non-repetitive combinations of two, three, and four sampling points selected from the nine sampling time points (predicted AUC = α + β_0_ C_0_ + β_1_C_1_ + … +β_n_C_n_).

The coefficient of determination (R^2^) of each prediction equation was calculated and compared to identify the most accurate model. The R^2^ value was used to identify the optimal regression equation by selecting those with R^2^ values >0.95. The validity and reliability of these equations were subsequently evaluated using validation datasets. For validation, error metrics, including the mean squared error (MSE), root mean squared error (RMSE), mean absolute error (MAE), and mean absolute percentage error (MAPE) were calculated and compared. MAPE values <10% were considered highly reliable, whereas those above 20% indicated the need for model improvement. Passing-Bablok regression and Bland–Altman analyses were employed to assess agreement and correlation between predicted and actual values (20, 21). Passing-Bablok regression analysis was used to confirm the monotonic relationship between the two variables. The Bland–Altman analysis is a method for evaluating the agreement between two measurement methods. It is used to compare a new measurement method with a standard measurement method and is suitable for visually evaluating bias (average difference) and measurement variability. Limits of agreement (LOA) were used to evaluate the agreement between the measurement methods, the narrower the limit, the higher the agreement between the measurement methods.

The standard deviation (SD) of differences is an index that indicates the degree of variability between measurement methods, the smaller the limit, the higher the agreement.

## Results

### Baseline characteristics

Baseline characteristics of the TAC-ER learning (n=48), TAC-ER validation (n=42), MPA learning (n=37), and MPA validation (n=43) datasets included in this study are summarized in **Tables 1** and **2**. For both TAC-ER and MPA, there were no significant differences in sex ratio, body mass index, dose per body weight, trough levels, or measured AUC between the learning and validation datasets.

**Table 1:** Clinical characteristics of renal transplant recipients (TAC-ER).

Parameters	Learning cohort	Validation cohort	*P* value
Patients, n	30	27	N/A
Number of TDM	48	42	N/A
Age at sampling (years)	44 ± 16.0	43 ± 14.7	1.00^##^
Male/female, n	27/21	21/21	0.55^#^
Weight (kg)	58 ± 10.7	54.3 ± 11.5	0.10^##^
BMI (kg/m^2^)	22 ± 2.9	21 ± 2.9	0.20^##^
Dose of TAC-ER (mg/kg)	0.1 ± 0.04	0.1 ± 0.04	0.50^##^
Trough of Tacrolimus (ng/ml)	5.5 ± 2.0	5.1 ± 2.2	0.34^##^
Actual AUC (ng · h/ml)	187 ± 58.6	182 ± 66.7	0.75^##^

Data presented as number or mean ± SD.

AUC, area under the curve; BMI, body mass index; MPA, mycophenolic acid; MMF, Mycophenolate mofetil, SD, standard deviation.

^#^Chi-square test ^##^Student's t-test.

**Table 2:** Clinical characteristics of renal transplant recipients (MPA).

Parameters	Learning sets	Validation sets	P value
Patients, n	31	20	N/A
Number of TDM	37	43	N/A
Age at sampling (years)	36 ± 16.2	29 ± 18.4	0.11^##^
Male/female, n	23/14	30/13	0.17^#^
Weight (kg)	55 ± 15.9	42 ± 19.1	0.1^##^
BMI (kg/m^2^)	21 ± 3.7	18 ± 3.1	0.08^##^
Dose of MMF (mg/kg)	24 ± 8.5	27 ± 9.8	0.23^##^
Trough of MPA (μg/ml)	2 ± 1.0	2 ± 1.6	0.61^##^
Actual AUC (μg · h/ml)	37 ± 15.3	39 ± 19.3	0.71^##^

Data presented as number or mean ± SD.

AUC, area under the curve; BMI, body mass index; MPA, mycophenolic acid; MMF, Mycophenolate mofetil, SD, standard deviation.

^#^Chi-square test ^##^Student's t-test.

### Overview of the pharmacokinetic profiles

**Figure 2:**
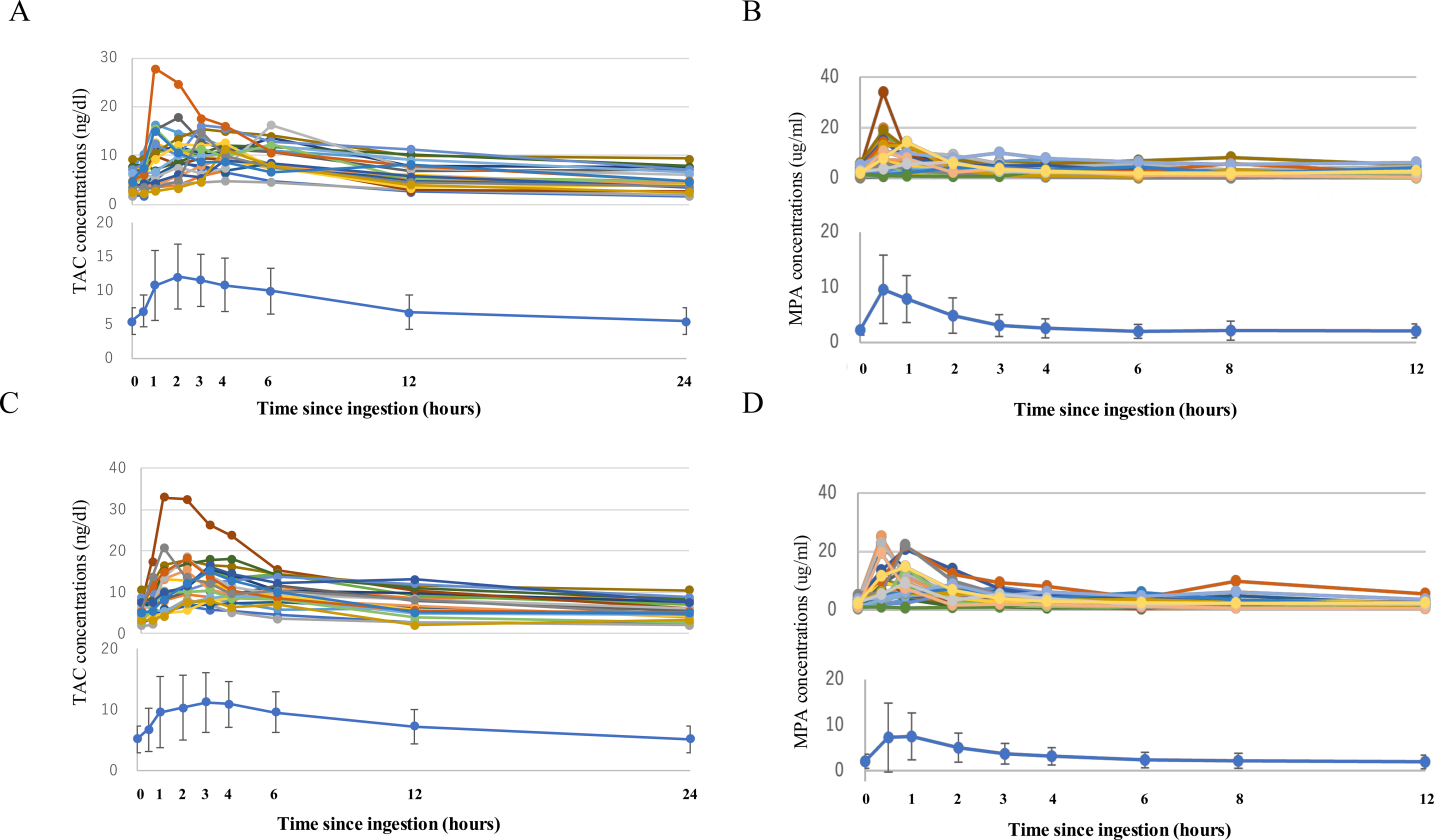
Pharmacokinetic profiles of TAC-ER and MPA. Overview of the pharmacokinetic profiles of the learning **(A**; TAC-ER and **B**; MPA**)** and validation **(C**; TAC-ER and **D**; MPA**)** datasets in patients undergoing renal transplantation. The upper graphs represent the pharmacokinetics of individual data, while the lower graphs show their mean ± standard deviation. TAC-ER, extended-release tacrolimus; MPA, mycophenolic acid.

Pharmacokinetic profiles of TAC-ER and MPA were divided into learning and validation datasets, respectively. In most patients treated with TAC-ER, blood concentrations stabilized within 4 h post-administration, with >60% reaching peak levels within 3 h (**Figures 2A, C**). MPA typically exhibits a biphasic pattern in blood concentration owing to enterohepatic circulation. However, in this study, most MPA blood concentrations reached their peak levels within 2 h, and the average (SD) plasma concentration did not provide sufficient data to clearly demonstrate a biphasic pattern (**Figures 2B, D**). The correlation between the single-point drug concentration and actual AUC was evaluated for TAC-ER and MPA in the learning datasets. TAC-ER showed the best correlation value at C_0_ (R^2^=0.759), followed by C_6_ (R^2^=0.672) and C_3_ (R^2^=0.618). For MPA, C_0_ was lower than that of TAC-ER (R^2^=0.497), whereas C_4_ demonstrated the best correlation (R^2^=0.649). Thus, the C_0_ single-point equation for TAC-ER demonstrated a relatively good correlation, whereas the predictive ability of MPA showed an inferior correlation (**Supplementary Table S1**).

### Selection of the best equations for TAC-ER AUC_0–24_ and MPA AUC_0-12_ in the learning datasets using limited sampling strategy

The optimal linear regression models for predicting the actual TAC-ER AUC_0–24_ and MPA AUC_0–12_ were selected based on the highest R^2^ values among all the models (**Supplementary Table S1**). **Table 3** summarizes the predictive performance of the equations that have a good correlation with a predictive AUC of R^2^ > 0.85.

**Table 3:** Correlation between predicted AUC calculated by various sampling time combination and actual AUC using linear regression analysis.

Sampling point	Sampling time	TAC-ER		MPA	
		R^2^	Model equations for AUC	R^2^	Model equations for AUC
Two Time Point Combinations	C_0_, C_6_	0.894	18.45+11.82*C_0_+10.35*C_6_	0.887	10.34+5.75*C_0_+6.98*C_6_
	C_0.5_, C_6_	0.85	8.40+6.09*C_0.5_+13.59*C_6_	0.887	10.00+0.71*C_0.5_+10.48*C_6_
	C_1_, C_6_	0.814	16.849+2.104*C_1_+14.728*C_6_	0.931	4.12+2.19*C_1_+8.18*C_6_
Three Time Point Combinations	C_0_, C_0.5_, C_6_	0.911	4.96+9.99*C_0_+4.60*C_0.5_+9.50*C_6_	0.923	3.67+5.98*C_0_+0.59*C_0.5_+7,44*C_6_
	C_0_, C_1_, C_3_	0.951	2.13+19.39*C_0_+1.91*C_1_+5.01*C_3_	0.916	-1.46+5.49*C_0_+1.80*C_1_+4.10*C_3_
	C_0_, C_1_, C_4_	0.959	5.63+18.15*C_0_+2.49*C_1_+5.05*C_4_	0.907	-1.76+4.57*C_0_+1.96*C_1_+5.35*C_4_
	* C_0_, C_1_, C_6_	0.958	2.85+13.26*C_0_+2.57*C_1_+8.34*C_6_	0.962	3.89+3.29*C_0_+1.51*C_1_+7.06*C_6_
	C_0_, C_2,_ C_6_	0.935	0.88+13.01*C_0_+3.40*C_2_+7.35*C_6_	0.901	6.24+4.52*C_0_+1.84*C_2_+5.90*C_6_
	C_0_, C_3,_ C_6_	0.937	0.40+10.86*C_0_+4.69*C_3_+7.29*C_6_	0.891	7.46+4.40*C_0_+2.86*C_3_+5.17*C_6_
	C_0_, C_4,_ C_6_	0.938	9.47+10.47*C_0_+4.26*C_4_+7.37*C_6_	0.888	8.07+5.05*C_0_+2.69*C_4_+5.17*C_6_
Four Time Point Combinations	* C_0_, C_0.5,_ C_1,_ C_6_	0.958	1.07+12.58*C_0_+1.25*C_0.5_+2.28*C_1_+8.35*C_6_	0.967	-0.70+5.05*C_0_+-0.12*C_0.5_+2.14*C_1_+5.66*C_6_
	C_0_, C_0.5,_ C_2,_ C_6_	0.944	-5.53+11.74*C_0_+2.84*C_0.5_+3.04*C_2_+7.15*C_6_	0.944	5.53+11.74*C_0_+2.84*C_0.5_+3.04*C_2_+7.15*C_6_
	C_0_, C_0.5,_ C_3,_ C_6_	0.947	-7.59+9.68*C_0_+3.25*C_0.5_+4.26*C_3_+6.96*C_6_	0.938	-7.59+9.68*C_0_+3.25*C_0.5_+4.26*C_3_+6.96*C_6_
	C_0_, C_0.5,_ C_4,_ C_6_	0.953	-1.37+9.00*C_0_+3.95*C_0.5_+3.92*C_4_+6.88*C_6_	0.93	-1.37+9.00*C_0_+3.95*C_0.5_+3.92*C_4_+6.88*C_6_
	C_0_, C_1,_C_2,_C_3_	0.955	2.23+19.64*C_0_+1.56*C_1_+1.01*C_2_+4.15*C_3_	0.917	2.23+19.64*C_0_+1.56*C_1_+1.00*C_2_+4.15*C_3_
	C_0_,C_1,_C_2,_C_4_	0.963	3.72+18.37*C_0_+1.51*C_1_+1.91*C_2_+3.97*C_4_	0.911	3.72+18.37*C_0_+1.51*C_1_+1.91*C_2_+3.97*C_4_
	* C_0_,C_1,_C_2,_C_6_	0.959	2.86+13.77*C_0_+1.387*C_1_+1.920*C_2_+7.508*C_6_	0.962	1.72+2.70*C_0_+1.31*C_1_+1.37*C_2_+6.24*C_6_
	C_0_,C_1,_C_3,_C_4_	0.959	2.88+18.05*C_0_+2.07*C_1_+2.44*C_3_+3.18*C_4_	0.92	0.57+12.72*C_0_+1.84*C_1_+2.80*C_3_+6.79*C_4_
	* C_0_,C_1,_C_3,_C_6_	0.962	0.57+12.72*C_0_+1.84*C_1_+2.80*C_3_+6.79*C_6_	0.967	1.69+2.22*C_0_+1.43*C_1_+2.54*C_3_+5.44*C_6_
	* C_0_,C_1,_C_4,_C_6_	0.969	-0.32+12.66*C_0_+2.16*C_1_+3.37*C_4_+6.09*C_6_	0.968	1.11+2.40*C_0_+1.57*C_1_+3.01*C_4_+5.03*C_6_
	C_0_,C_2,_C_3,_C_6_	0.947	1.44+13.07*C_0_+2.04*C_2_+2.37*C_3_+6.60*C_6_	0.902	6.09+4.26*C_0_+1.33*C_2_+1.28*C_3_+5.39*C_6_
	C_0_,C_2,_C_4,_C_6_	0.954	0.43+13.07*C_0_+2.46*C_2_+2.75*C_4_+5.92*C_6_	0.902	5.97+4.43*C_0_+1.60*C_2_+0.94*C_4_+5.40*C_6_

*Equations with R^2^ values > 0.95 for both TAC-ER and MPA AUC, area under the concentration curve; MPA, mycophenolic acid; TAC-ER, extended-release tacrolimus.

C_0_ is easily obtainable for evaluating intra-individual drug fluctuations in outpatient settings. C_0_ has also been reported to have a strong correlation with the actual AUC, particularly for TAC-ER; therefore, it was included in all selected equations (4). Clinically feasible sampling times for outpatient use were limited to within 6 h after administration, with a maximum of four sampling points, allowing drug concentration measurements in an outpatient setting.

The equations that included C_0_ did not show inferior R^2^ values compared with those without C_0_. Predictive equations incorporating multiple sampling points outperformed the single-point equations. Among the three-point models, C_0_-C_1_-C_6_ (TAC-ER: R^2^ = 0.958; MPA: R^2^ = 0.962) exhibited the highest R^2^ values. For four-point models, C_0_-C_1_-C_2_-C_6_ (TAC-ER: R^2^ = 0.959, MPA: R^2^ = 0.962), C_0_-C_1_-C_3_-C_6_ (TAC-ER: R^2^ = 0.962, MPA: R^2^ = 0.967), and C_0_-C_1_-C_4_-C_6_ (TAC-ER: R^2^ = 0.969, MPA: R^2^ = 0.968) demonstrated superior results to the other four-points model. Therefore, these four prediction models were used to validate the datasets.

### Limited sampling with C_0_-C_1_-C_3_-C_6_ showed the best predictive performance for TAC-ER AUC_0–24_ and MPA AUC_0–12_ in the validation datasets

**Figure 3:**
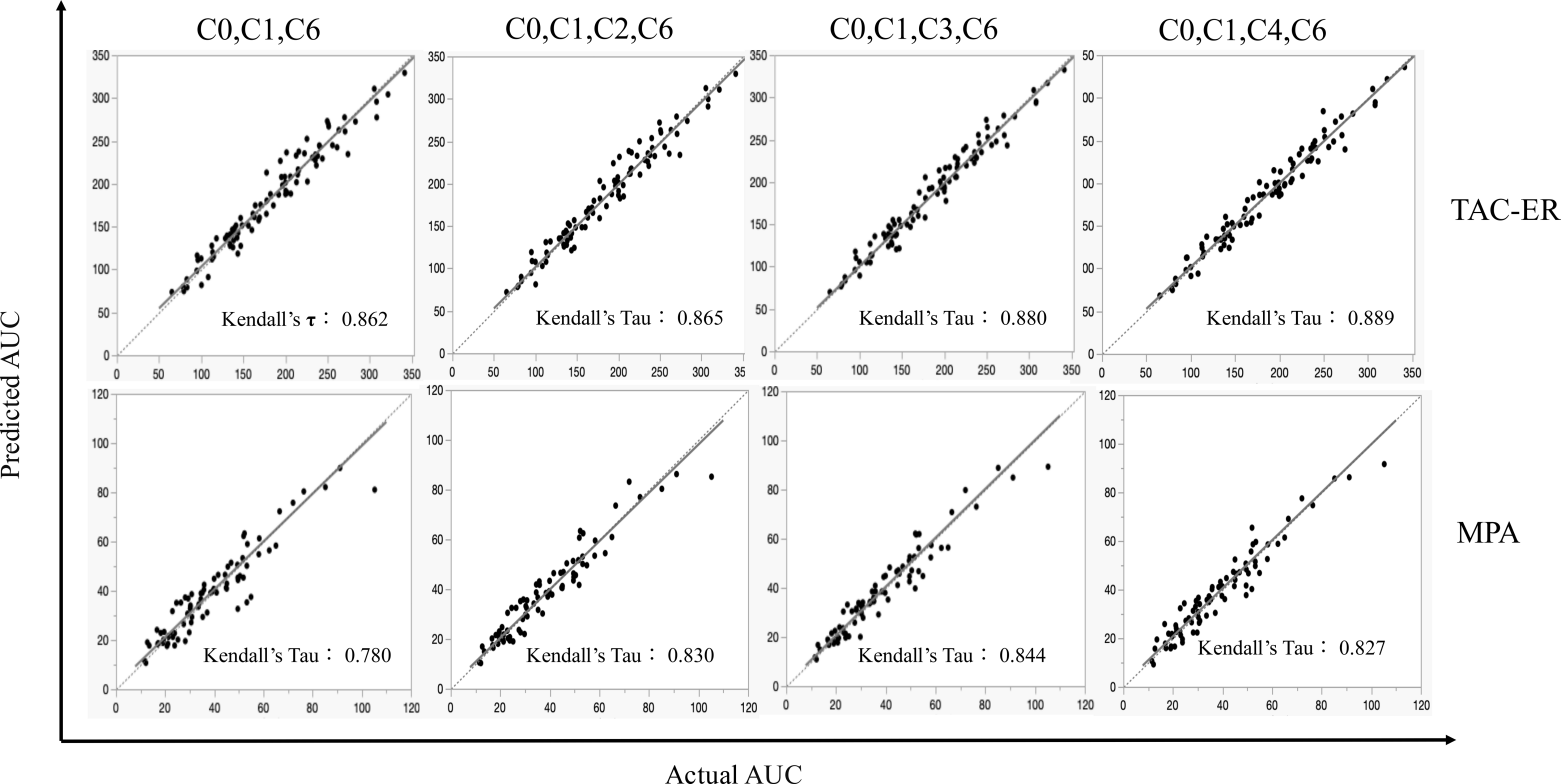
Passing-Bablok regression analysis comparing predicted AUC with actual AUC. The solid line represents the regression line and the dashed line indicates the line of identity (slope = 1). This analysis assessed the agreement between predicted and observed values. AUC, area under the curve.

**Figure 4:**
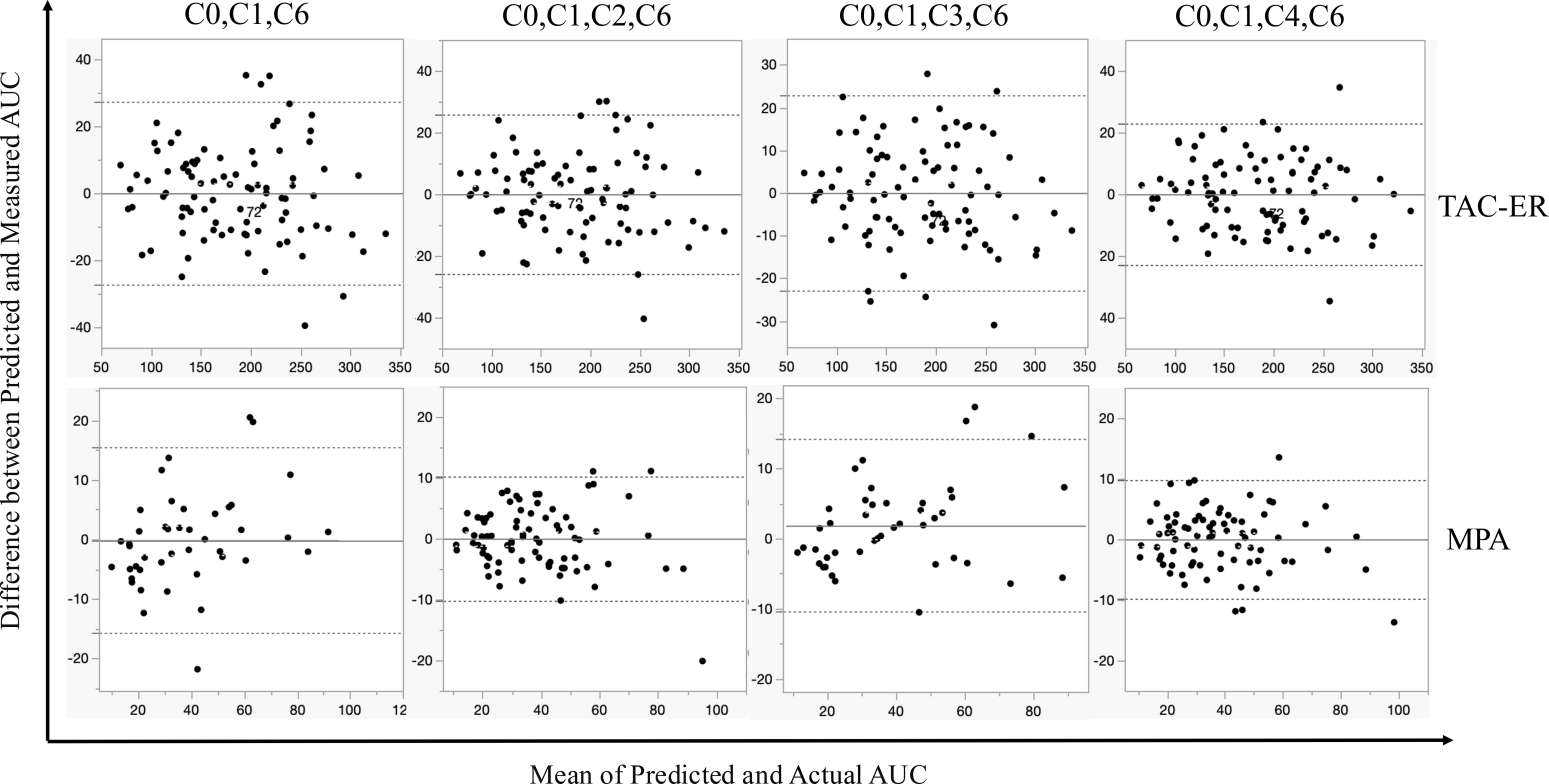
Bland–Altman plots comparing predicted AUC with actual AUC. The dotted lines represent 95% upper and lower limits. The solid line represents an average of the arithmetic differences between predicted AUC and measured AUC. AUC, area under the curve.

We applied the equations to the validation datasets and evaluated them using MSE, RMSE, MAE, MAPE, Kendall’s τ derived from Passing-Bablok regression analysis, and LOA and SD of differences derived from the Bland–Altman analysis (**Table 4**). In the Passing-Bablok regression plot, all the equations showed good agreement with a regression line slope close to 1 and an intercept near 0 (**Figure 3**). All the four models performed similarly for TAC-ER, whereas for MPA, the C_0_-C_1_-C_6_ model yielded a relatively lower value (Kendall’s τ = 0.780). In the Bland–Altman plot, no significant differences were observed between the predicted and actual AUC values with the LOA, including a 95% difference (**Figure 4**).

**Table 4:** Prediction performance evaluated on validation data sets with limited sampling formulas for TAC-ER and MPA AUCs elected in learning data sets.

Therapeutic Drug	Sampling point	R2	Prediction error			
			MSE	RMSE	MAE	MAPE(%)*
TAC-ER	Co,C_1_,C_6_	0.958	195.66	13.988	11.001	6.39
	C_0_,C_1_,C_2_,C_6_	0.959	171.93	13.112	10.29	5.905
	Co,C1,C3,C6	0.962	134.89	11.614	9.384	5.464
	Co,C1,C4,C6	0.969	136	11.662	9.392	5.444
MPA	Co,C_1_,C_6_	0.962	40.582	6.37	4.712	13.485
	Co,C1,C2,C_6_	0.962	69.837	8.357	6.665	20.04
	Co,C1,C3,C_6_	0.967	24.147	4.914	3.778	10.284
	Co,C1,C4,C6	0.968	24.823	4.982	3.946	12.082

*MAPE values below 10% are considered highly reliable, while values above 50% indicate the need for model improvement.

CI, Confidence Interval; LOA, Limits of Agreement; MAE, mean absolute error; MAPE, mean absolute percentage error; MD, Mean Difference; MPA, mycophenolic acid; MSE, mean squared error; RMSE, root mean squared error; SD, Standard Deviation; TAC-ER, extended-release tacrolimus.

For TAC-ER, all the four models demonstrated comparable predictive performance (**Table 4**).

For MPA, C_0_-C_1_-C_3_-C_6_ demonstrated the best performance values in all the parameters (R^2^ = 0.967, MSE = 24.147, RMSE = 4.914, MAE = 3.778, MAPE = 10.284%, and Kendall’s τ = 0.844).

Therefore, there was no clear superiority among the four combinations in TAC-ER; however, the C_0_-C_1_-C_3_-C_6_ combination was considered the best in MPA. Based on the above, it was thought that the C_0_-C_1_-C_3_-C_6_ combination was the ideal combination of blood collection points in terms of balancing the burden on the patient and accuracy in the simultaneous LSS of TAC-ER and MPA.

## Discussion

Appropriate therapeutic monitoring of immunosuppressive agents is crucial in preventing serious complications such as opportunistic infections due to over-immunosuppression and acute rejection episodes resulting from inadequate drug exposure. While determination of the actual AUC through complete pharmacokinetic profiling would provide the most accurate assessment of drug exposure, obtaining 9 blood samples at multiple time points over 12–24 hours from all patients is impractical in routine clinical practice. Such intensive sampling protocols impose substantial logistical burdens on both patients and healthcare staff, making it unfeasible to implement in everyday transplant management. Therefore, developing a limited sampling strategy (LSS) that maintains adequate predictive accuracy while requiring only 3–4 blood samples obtainable during a single outpatient visit is of considerable clinical importance for optimizing personalized immunosuppressive therapy in the ambulatory care setting.

While many centers administer MMF at a fixed dose, many reports have highlighted the importance of monitoring drug exposure using AUC to achieve individualized therapy. Accordingly, MPA exposure is assessed via MPA-AUC in the United States, Europe, China, and Japan, following regional guidelines and recommendations (14–18). Similarly, therapeutic drug monitoring of TAC, particularly once-daily extended-release TAC (TAC-ER), remains essential to ensure adequate and personalized immunosuppressive therapy (9, 19).

We developed and compared predictive equations for TAC-ER and MPA AUCs using various combinations of available sampling time points. Among them, we selected four combinations of sampling points in which the prediction equations using a combination of C_0_-C_1_-C_6_, C_0_-C_1_-C_2_-C_6_, C_0_-C_1_-C_3_-C_6_, and C_0_-C_1_-C_4_-C_6_, demonstrated sufficient predictive accuracy. Although the predictive accuracy improves with additional sampling points, our findings indicate that a clinically feasible number and timing of samples still achieve a sufficiently high predictive performance. There was no clear superiority among the four combinations in TAC-ER; however, the C_0_-C_1_-C_3_-C_6_ combination was considered the best in MPA. In the validation datasets, predictive assessments demonstrated that for both TAC-ER and MPA, the C_0_-C_1_-C_3_-C_6_ combination consistently achieved the highest accuracy across all metrics. The combination of blood sampling times can simultaneously measure the AUC_0−24_ for once-daily TAC-ER and AUC_0−12_ for twice-daily MMF, which have different drug profiles. While it is scientifically reasonable to construct an AUC estimation formula including C_12_, in actual clinical practice, TAC and MPA concentrations measurement at night (7pm or 8pm) is required not only for inpatients but also for outpatients. Thus, we prioritized a combination of blood sampling points applicable in both inpatient and outpatient settings. Note that the formulas including C_12_ are listed in **Supplementary Table S1** along with their correlation coefficients. Any of the correlation coefficient (R^2^) for the formulae including C_12_ showed no significant improvement compared to formulae using C_0_, C_1_, C_3_, and C_6_. To ensure clinical feasibility in an outpatient setting, we determined that 4 blood concentrations over a 6-hour period represent the optimal approach. As stated in the manuscript, these constraints are intended to minimize the burden on patients and medical staffs while maintaining practicality. A more extensive (longer) sampling period could potentially improve accuracy of estimation of AUC but is impractical for routine clinical care. The selected model demonstrated high predictive performance (R^2^ > 0.95) even within this limited sampling framework. Conversely, inclusion of C6 is essential to maintain model accuracy. As shown in **Supplementary Table S1**, omitting C_6_ and constructing the predictive formula using only sampling points within a shorter 4-hour window results in reduced predictive performance. As MPA undergoes enterohepatic circulation, it is recommended that at least one blood sampling point after six hours be included for accurate AUC estimation (22). Taking this into account, our formula is also reasonable.

To the best of our knowledge, this is the first study to report the development and validation of an LSS using regression equations to predict the AUC_0–24_ of TAC-ER and AUC_0–12_ of MPA. Furthermore, this study included patients with varying postoperative periods, sex, and age, ensuring that the developed equation is applicable to diverse patient backgrounds.

TAC-ER, an immunosuppressive drug containing TAC as its active ingredient, was developed by Astellas Pharma in Japan (23, 24). Compared with Prograf^R^, which requires twice-daily dosing, TAC-ER produces a more gradual peak in blood concentration after post-administration (23). This reduces the risk of side effects associated with fluctuations in blood levels, thereby enhancing patient safety and tolerance (25–27). MMF is converted to MPA in the body and metabolized as a glucuronide conjugate in the liver (28, 29). MPA is characterized by a complex metabolism and is significantly affected by enterohepatic recirculation, leading to a biphasic blood concentration profile and considerable interindividual variability (30). As for sample size in the current study, testing Kendall’s correlation coefficient revealed that even with a correlation coefficient of 0.8 and 20 cases, the power exceeded 98%. Although the retrospective nature of the study inherently imposed limitations on sample size, the results obtained demonstrate sufficient power, and the sample size is considered adequate.

This study has some limitations. All cases were taking TAC and MMF orally. However, blood sampling did not measure two drug concentrations simultaneously from the same patients; instead, the datasets were collected from the clinical records where only TAC was measured or where only MPA was measured. This study utilized these datasets. TAC and MMF/MPA have totally different metabolism pathways dependent on CYP3A4/5 (1) and UGT2B7 (28), respectively. Although the TAC and MMF were administered independently, it is considered that measuring the concentrations of the two drugs independently should pose little issue. However, potential pharmacokinetic interactions between the two drugs might not be excluded. Further validation in patients receiving both drugs is warranted. Secondly, this study included multiple AUC measurements obtained from the same subjects at different time points. Consequently, it cannot be ruled out that disparities in contribution may have arisen during the creation of prediction models across individual patients. On the other hand, it may also have enabled the development of estimation models capable of addressing diverse post-transplant periods. Therefore, further validation within a more controlled prospective cohort is desirable. Future studies should evaluate clinical outcomes and assess how post-transplantation periods and patient conditions affect the accuracy of the prediction equations in more-controlled cohort prospectively.

In conclusion, this study demonstrated that LSS using C_0_-C_1_-C_3_-C_6_ reliably and accurately estimated both the actual TAC-ER AUC_0–24_ and MPA AUC_0–12_ in kidney transplant recipients. LSS using C_0_-C_1_-C_3_-C_6_ can be feasibly implemented in outpatient clinical settings to reduce time and cost. The LSS algorithms validated in this study have proven to be highly useful tools for the TDM of TAC-ER and MPA exposure in clinical settings.

## Data Availability

The raw data supporting the conclusions of this article will be made available by the authors, without undue reservation.
